# COMP report: CPQR technical quality control guidelines for accelerator‐integrated cone‐beam systems for verification imaging

**DOI:** 10.1002/acm2.12302

**Published:** 2018-03-06

**Authors:** Jean‐Pierre Bissonnette

**Affiliations:** ^1^ Department of Radiation Oncology University of Toronto Toronto ON Canada; ^2^ Department of Medical Physics Princess Margaret Cancer Centre Toronto ON Canada

**Keywords:** cone‐beam CT, quality control guidelines, radiation treatment therapy equipment

## Abstract

The Canadian Organization of Medical Physicists, in close partnership with the Canadian Partnership for Quality Radiotherapy has developed a series of Technical Quality Control (TQC) guidelines for radiation treatment equipment. These guidelines outline the performance objectives that equipment should meet in order to ensure an acceptable level of radiation treatment quality. The TQC guidelines have been rigorously reviewed and field tested in a variety of Canadian radiation treatment facilities. The development process enables rapid review and update to keep the guidelines current with changes in technology. This article presents the quality control guideline accelerator‐integrated cone‐beam systems for verification imaging that has resulted from this process.

## 1 INTRODUCTION

The Canadian Partnership for Quality Radiotherapy (CPQR) is an alliance among the three key national professional organizations involved in the delivery of radiation treatment in Canada: the Canadian Association of Radiation Oncology, the Canadian Organization of Medical Physicists, and the Canadian Association of Medical Radiation Technologists. Financial and strategic backing is provided by the federal government through the Canadian Partnership Against Cancer, a national resource for advancing cancer prevention and treatment. The mandate of the CPQR is to support the universal availability of high quality and safe radiotherapy for all Canadians through system performance improvement and the development of consensus‐based guidelines and indicators to aid in radiation treatment program development and evaluation. The development of the individual Technical Quality Control (TQC) guidelines is spearheaded by expert reviewers and involves broad stakeholder input from the medical physics and radiation oncology community.^1^

This document contains detailed performance objectives and safety criteria for *Accelerator‐Integrated Cone‐Beam Systems for Verification Imaging*. Please refer to the overarching document *Technical Quality Control Guidelines for Canadian Radiation Treatment Centres* 2 for a programmatic overview of technical quality control, and a description of how the performance objectives and criteria listed in this document should be interpreted.

All information contained in this document is intended to be used at the discretion of each individual center to help guide quality and safety program improvement. There are no legal standards supporting this document; specific federal or provincial regulations and license conditions take precedence over the content of this document.

## 2 SYSTEM DESCRIPTION

In this report, a linac integrated cone‐beam CT (CBCT) imaging system is defined as a kV source and a flat panel x‐ray detector that are attached orthogonally to a linear accelerator (kV‐CBCT). Unlike conventional CT, kV‐CBCT uses a cone shaped x‐ray beam and acquires an entire volume (14–26 cm in length) in a single gantry rotation lasting approximately 2 min. To acquire the kV‐CBCT projection data, flat‐panel detectors are used in fluoroscopy mode, obtaining multiple projections per second; these projections are used to reconstruct the CBCT volumetric images. The imaging system is capable of providing radiographic, fluoroscopic, and CBCT imaging capabilities for image‐guided radiation therapy, and possibly simulation. kV‐CBCT produces a full CT dataset that, although below diagnostic quality, is generally adequate for directly targeting bone and, in some sites, soft tissue. At this writing, two commercial systems are available in Canada: the On‐Board Imager™ (OBI) by Varian Medical Systems, Inc. (Palo Alto, CA, USA), and the Elekta XVI system by Elekta Oncology Systems (Stockholm, Sweden).

A variant commercial offering from Siemens uses similar principles, but uses the linear accelerator as the imaging x‐ray source and an optimized portal imaging system for CBCT image acquisition and reconstruction.

All systems can produce two‐dimensional (2D) images that can be registered with reference digitally reconstructed radiographs generated by treatment planning systems and three‐dimensional (3D) datasets that can be aligned with the planning CT. Both the 2D and 3D approaches allow verification and correction of patient positioning prior to delivery of the therapeutic dose.

Various attempts to recommend quality control guidelines for accelerator‐integrated cone‐beam systems have been reported in the literature and have been considered in developing the current guidelines shown in Tables 1, 2, 3.^3^, ^4^, ^5^, ^6^, ^7^, ^8^, ^9^, ^10^, ^11^, ^12^, ^13^, ^14^

**Table 1:** Daily quality control tests

Designator	Test	Performance
		Action
*Daily*		
DS1	Collision and safety interlocks	Functional
DS2	Laser/image/treatment isocenter coincidence; *or*	±2 mm
	Phantom localization and repositioning with couch shift	±2 mm
DS3	Warm‐up: X‐ray tube and flat panel operation	Functional
DS4	Database integrity and software operation	Functional

**Table 2:** Monthly quality control tests

Designator	Test	Performance
		Action
*Monthly*		
MS1	Geometric calibration maps; *or*	Replace/refresh; ±0.25 mm
	kV/MV/laser alignment	±1 mm
MS2	End‐to‐end test, including couch shift accuracy	±1 mm
MS3	Image quality: spatial integrity	Reproducible
MS4	Image quality: uniformity, noise	Reproducible
MS5	Image quality: low‐contrast visibility	Reproducible
MS6	Image quality: high‐contrast resolution	≤2 mm (or ≤5 lp/cm)
MS7	Image quality: CT number accuracy and stability	Reproducible
MS8	Records	Complete

**Table 3:** Annual quality control tests

Designator	Test	Performance
		Action
*Annual*		
AS1	Radiation dose	Reproducible
AS2	X‐ray generator performance	Reproducible
AS3	Orientation	Reproducible
AS4	System operation: disk space and IT infrastructure	Functional
AS5	Independent quality control review	Complete

## 3 RELATED TECHNICAL QUALITY CONTROL GUIDELINES

In order to comprehensively assess accelerator‐integrated cone‐beam systems performance, additional guideline tests, as outlined in related CPQR TQC guidelines must also be completed and documented, as applicable:^15^

- Safety systems
- Medical linear accelerators and multileaf collimators
- Major dosimetry equipment

## 4 TEST TABLES

**Table:** Notes on daily tests

DS1	As per the manufacturer's recommendations. Variations exist between manufacturers.
DS2	Phantom localization and repositioning tests can be performed using dedicated phantoms that offer orientation features or simple ball bearings. An accuracy of ±2 mm has been published for this test.^4^
DS3	The x‐ray tube warm‐up procedure should follow the manufacturer's instructions. These quality control tests are typically integrated within the procedure for DS2.
DS4	Software does not crash during test acquisition, and sufficient disk space is available for the day's operation. Digital Imaging and Communications in Medicine (DICOM) links to and from treatment planning system and picture archiving and communication system (PACS) should be functional.
	These quality control tests are typically integrated within the procedure for DS2.

**Table:** Notes on monthly tests

MS1	The geometric calibration procedure should follow the manufacturer's instructions. Depending on user experience and data demonstrating stability of geometric calibration, frequency of testing may be relaxed to biannually or upon service/upgrade, whichever occurs first.
MS2	End‐to‐end test of the image‐guidance procedure using rigid phantoms. A reference CT scan of the phantom is required.
MS3–6	Image quality control tests results can be extracted from a single image acquisition of a standard CT image quality phantom. Manufacturers typically supply such phantoms as part of the purchase. Users are strongly recommended to follow exactly the instructions from the manufacturer's Customer Acceptance Documents.
MS7	Image quality control tests results can be extracted from a single image acquisition of a standard CT image quality phantom. Manufacturers typically supply such phantoms as part of the purchase. Users are strongly recommended to follow exactly the instructions from the manufacturer's Customer Acceptance Documents.
	Depending on user experience and data demonstrating stability of these quality control metrics, frequency of testing may be relaxed to biannually or upon service/upgrade, whichever occurs first.
	Perform only if the clinic uses such images for treatment planning and dose calculations performed with heterogeneity corrections. This should be tested only for those validated techniques used clinically.
MS8	Documentation relating to the daily quality control checks, preventive maintenance, service calls, and subsequent checks must be complete, legible, and the operator identified.

**Table:** Notes on annual tests

AS1	Point dose measurements using a Farmer ion chamber calibrated for orthovoltage energies. Suitable points would be representative of axial and skin doses. See Osei et al. (2009)^16^ for details.
AS2	For kV‐CBCT systems only. As for any x‐ray tube used clinically, tube kVp, half value layers (HVLs), mAs linearity, and accuracy of time and mA should be verified for those tube settings used by the CBCT system. Provincial regulations may supersede the baseline tolerances.
AS3	Using a phantom with asymmetrical features (e.g., anthropomorphic phantom or daily quality assurance phantom), compare a CBCT image with reference images in terms of orientation (i.e., anterior/posterior, superior/inferior, left/right directions). Also, verify that CT images obtained with the phantom in prone or supine positions, or scanned head first or feet first, are suitably transmitted to the CBCT system.
AS4	The clinic is encouraged to have a documented protocol for image archival. This protocol would specify how long files are kept in the clinical database, whether raw projections are stored or not, the pixel size of stored 3D datasets, and archival protocols and frequencies to offline disk systems or PACS.
AS5	To ensure redundancy and adequate monitoring, a second qualified medical physicist must independently verify the implementation, analysis, and interpretation of the quality control tests at least annually.

## CONFLICT OF INTEREST

The author has no conflict of interest to declare.
